# Expectation propagation for large scale Bayesian inference of non-linear molecular networks from perturbation data

**DOI:** 10.1371/journal.pone.0171240

**Published:** 2017-02-06

**Authors:** Zahra Narimani, Hamid Beigy, Ashar Ahmad, Ali Masoudi-Nejad, Holger Fröhlich

**Affiliations:** 1 Laboratory of Systems Biology and Bioinformatics (LBB), Institute of Biochemistry and Biophysics, University of Tehran, Tehran, Iran; 2 Department of Computer Engineering, Sharif University of Technology, Tehran, Iran; 3 Algorithmic Bioinformatics, Bonn-Aachen International Center for IT, University of Bonn, Bonn, Germany; 4 UCB Biosciences GmbH, Monheim, Germany; Tampere University of Technology, FINLAND

## Abstract

Inferring the structure of molecular networks from time series protein or gene expression data provides valuable information about the complex biological processes of the cell. Causal network structure inference has been approached using different methods in the past. Most causal network inference techniques, such as Dynamic Bayesian Networks and ordinary differential equations, are limited by their computational complexity and thus make large scale inference infeasible. This is specifically true if a Bayesian framework is applied in order to deal with the unavoidable uncertainty about the correct model. We devise a novel Bayesian network reverse engineering approach using ordinary differential equations with the ability to include non-linearity. Besides modeling arbitrary, possibly combinatorial and time dependent perturbations with unknown targets, one of our main contributions is the use of Expectation Propagation, an algorithm for approximate Bayesian inference over large scale network structures in short computation time. We further explore the possibility of integrating prior knowledge into network inference. We evaluate the proposed model on DREAM4 and DREAM8 data and find it competitive against several state-of-the-art existing network inference methods.

## Introduction

Cellular components function through their interaction in form of biological networks, such as regulatory and signaling pathways [1]. With the advances of experimental methods and the emergence of high-throughput techniques, such as DNA microarray and next generation sequencing, the measurement of expression values of genes on whole genome scale is now possible. These advances have motivated attempts to learn molecular networks from experimental data. However, network inference from experimental data is computationally nontrivial, because the number of variables (typically genes, or proteins) usually exceeds the number of samples. Moreover, the number of possible network structures increases super-exponentially with the number of network nodes. Therefore the search space to look for the true network is very large even for small graph instances and thus prevents the use of exact methods.

With the emergence of targeted perturbation techniques such as RNAi [2] and more recently CRISPR-Cas9 [3], it becomes possible to study the effect of specific gene silencing on a whole molecular network in a systematic manner, thus enabling identification of causal networks based on multiple intervention effects. Based on this fact, several groups of researchers have focused their work on network learning from perturbation data.

A large number of network computational methods exist, ranging from purely graph based [4–6] over Bayesian Networks [7–12], factor graphs [13] and epistasis analysis [14] to mechanistic models [15, 16]. Moreover, specifically for high-dimensional indirect perturbation effects (Dynamic) Nested Effects Models [16–18] have been proposed.

In this work, we follow a mechanistic ODE modelling framework such as one described in [15, 19–21]. Molinelli et al. described an efficient inference method based on Belief Propagation (BP) and showed that their method has similar performance as exact Bayesian inference [16]. That method relies on a steady state assumption and searches over a finite set of edge weights in order to ensure convergence. The required data discretization is a principal limitation of the method and potential source of error in a practical application. Furthermore, the steady state assumption is only valid in specific applications, excluding time series data.

In contrast, the proposed method called FBISC (Fast Bayesian Inference of Sparse Causal networks), neither requires any data discretization step nor does it rely on a steady state assumption. It can be applied to time series as well as steady state data. Akin to Molinelli et al. we allow for modeling non-linear regulatory relationships between molecules. To enable causal inference we allow for arbitrary, possibly combinatorial interventions. Our method is Bayesian and thanks to the employed Expectation Propagation (EP) scheme [22] computationally attractive.

Sometimes prior information is available about the structure of the network we wish to infer. The Bayesian framework used in our method allows us to incorporate prior knowledge into the network inference procedure in a natural and flexible way. This is achieved via a “spike and slab” prior [23], which at the same time enforces sparsity of the network. The spike and slab prior is known to yield less biased estimates than lasso-type L1 penalties, as e.g. employed in the “Inferelator” [24].

## Materials and methods

### 2.1 Modeling perturbations and dynamics

To ease the representation of our model, we first assume we have time-series data. The case of steady state data will be explained later. Let *Y* = {*Y*_1_,*Y*_2_,…,*Y*_*n*_} be the molecules (genes, proteins, etc.) for which we have available measurements at different time points (*t*). We would like to estimate the unknown network topology underlying their interaction. The time-series dataset can be described as $D=\{y_{ir}^{c}(t)|i=1,\ldots ,n,r=1,\ldots ,q_{i},c=1,\ldots ,m\}$, where q_*i*_ is the number of replicate measurements for molecule *i*, and *m* is the number of perturbations. *C* = {1,2,…,m} represents the set of all perturbation experiments, in which each *c* ∈ *C* can either directly influence one molecule or a subset of molecules *Y*_*c*_ ⊆ *Y*, i.e. perturbations can be targeted or combinatorial.

**Fig 1:**
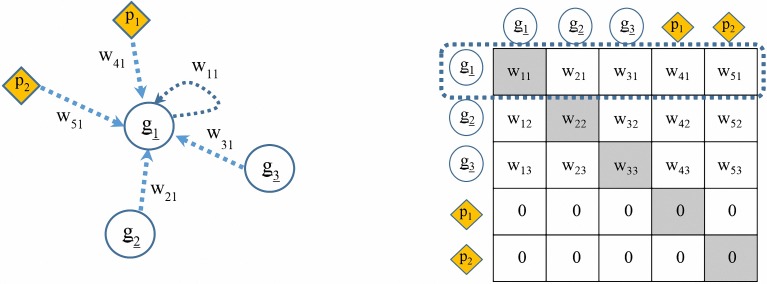
An example network of 3 genes (g_1_, g_2_, g_3_) and 2 perturbation nodes (p_1_, p_2_) and its related weight matrix. The goal is to infer network edges (w elements). The matrix is interpreted in the sense that elements in each row represent the weights of all incoming edges to a specific node (here: g1). The last two rows are zero, because we do not have edges directed towards perturbation nodes.

We have to take into account that perturbations may not exhibit the same quantitative effect on all directly influenced molecules. For example, we can think *c* to consist of a treatment with two ligands *A* and *B*. At given concentrations *A* might strongly inhibit protein *P1*, whereas *B* might exhibit a moderate effect on protein *P2*. In our model, we capture this behavior by including a set of extra nodes in our network, and a set of extra edges connecting perturbations with perturbed molecules. All the edges in our network are weighted, and with our model, we will infer the difference in the perturbation strength on different target molecules based on data. In conclusion, our network is a graph Γ = (Y∪C,*ε*), with the set of nodes Y∪C and set of edges ϵ. The set of edges for this graph consists of all possible connections between regular nodes (i.e. all nodes except the perturbation nodes) and also the connections from our perturbation nodes to our regular nodes. So, our adjacency matrix to be inferred is a *(n+m)×(n+m)* weight matrix *W* = (*w*_*ij*_). We do not aim to infer any edge pointing to the perturbation nodes. By means of this representation we are able to model interactions between our network nodes (genes, proteins), and also between perturbation nodes and their targets as shown in Fig 1.

In general *c* ∈ *C* can be time dependent, represented as *x*_*c*_*(t)*, which can be Boolean (1 indicating perturbation, 0 no perturbation) or fully quantitative. A special case is when *x*_*c*_*(t) = 1* for all *t = 1*,*…*,*T*. Targets of perturbation nodes do not have to be fully known; they can be inferred from data with our method. The technique for that (Expectation Propagation) is described later.

We can in principle model perturbations either as perfect (ideal) interventions or as soft interventions: Perfect interventions remove the influence of any other on the perturbed node, whereas soft interventions just increase the probability of the target node to be perturbed [25]. By using explicit intervention nodes and correspondingly weighted edges we here rely on a soft intervention scheme, which we believe to be closer to biological reality.

We assume an ordinary differential equation system (ODE) for describing the dynamics of the molecular network relative to known interventions:

$$\frac{dy}{dt}=f(y(t),x_{c}(t))$$ (1)

Function f can be linear or non-linear [26]. Linear ODEs are not capable of capturing important biological phenomena such as coupled perturbation effects, nonlinear interactions, and switch-like behavior [15]. Inspired by [15], we thus propose the following approach for representing the time dependent behavior of system measurements, $\left\{y_{ir}^{c}(t)\right\}$, via a set of coupled non-linear differential equations:

$$\frac{d}{dt}y_{ir}^{c}=\beta_{i}\frac{1}{1+\exp(-\sum_{p\neq i}w_{pi}z_{pr}^{c}(t))}-\alpha_{i}y_{ir}^{c}(t)$$ (2)

Here $z_{pr}^{c}(t)$ represents the time series of a measurement or perturbation node. The upper and lower bounds of $\frac{d}{dt}y_{ir}^{c}$ are controlled by positive parameters *α*_*i*_ and *β*_*i*_.

Eq (2) can be linearly approximated as

$$\frac{d}{dt}y_{ir}^{c}\approx \frac{y_{ir}^{c}(t+1)-y_{ir}^{c}(t)}{\Delta t}$$ (3)

yielding

$$y_{ir}^{c}(t+1)=\Delta_{t}\beta_{i}\frac{1}{\underset{≔R_{ir}^{c}(t)}{\underset{︸}{1+\exp(-\sum_{p\neq i}w_{pi}z_{pr}^{c}(t))}}}+y_{ir}^{c}(t)[1-\alpha_{i}\Delta_{t}]$$ (4)

where Δ_t_ is the length of the known time interval between subsequent measurements t and t+1. There is no constraint on equality of interval lengths. Δ_t_ weights the influence of measurements at time t on those at time point *t+1*. Shorter time intervals increase the influence of $y_{ir}^{c}(t)$ and decrease the effect of $R_{ir}^{c}(t)$.

Under steady state conditions we have $\frac{d}{dt}y_{ir}^{c}=0$ and hence we obtain:

$$y_{ir}^{c}=\beta_{i}\frac{1}{1+\exp(-\sum_{p\neq i}w_{pi}z_{pr}^{c})}.\frac{1}{\alpha_{i}}$$ (5)

Notably, we have removed the dependency on time *t* in the formula here due to the steady state condition.

### 2.2 Bayesian model fitting

#### 2.2.a Likelihood model

Let $\mu_{i}(t)≔\Delta_{t}\beta_{i}R_{ir}^{c}(t)+y_{ir}^{c}(t)[1-\alpha_{i}\Delta_{t}]$ for i = 1,…,n. Furthermore, let σ_i_ denote the Gaussian measurement noise for molecule *i*. The likelihood of measured data *D* given weight matrix *W* and known measurement noise can then be written as:

$$p(D|W,\sigma^{2})=\prod_{c\in C}\prod_{t=1}^{T-1}\prod_{i=1}^{n}\prod_{r=1}^{q_{i}}N(y_{ir}^{c}(t+1)|\mu_{i}(t),\sigma_{i})$$

Typically the number of replicate measurements *q*_*i*_ per molecule is small, and thus the empirical variance is an unreliable estimate of the true $\sigma_{i}^{2}$ In order to account for this fact we assume the true noise variance $\sigma_{i}^{2}$ to be drawn from an inverse gamma distribution:

$$\sigma_{i}^{2}∼IG(a,b)$$

With this setting the *marginal* likelihood $p(y_{ir}^{c}(t)|\mu_{i}(t))$, integrating out the unknown $\sigma_{i}^{2}$, can be computed in closed analytical form [27]:

$$p(y_{ir}^{c}(t+1)|\mu_{i}(t))=\frac{\Gamma (a+\frac{1}{2})}{\Gamma (a)}\frac{1}{{\left(2\pi b\right)}^{\frac{1}{2}}}\frac{1}{{\left(1+\frac{1}{2b}{\left(\mu_{i}(t)-y_{ir}^{c}(t+1)\right)}^{2}\right)}^{a+\frac{1}{2}}}$$

Accordingly, the marginal likelihood of the data given weight matrix *W* is given by

$$p(D|W)=\prod_{c\in C}\prod_{t=1}^{T-1}\prod_{n=1}^{n}\prod_{r=1}^{q_{i}}p(y_{ir}^{c}(t+1)|\mu_{i}(t))$$ (6)

Notably, using Eq (5) in the steady state situation Eq (6) changes into:

$$p(D|W)=\prod_{c\in C}\prod_{i=1}^{n}\prod_{r=1}^{q_{i}}p(y_{ir}^{c}|\mu_{i})$$ (7)

Note that we dropped *t* here to make the independence of time explicit.

In our method we use Eq (6) and Eq (7) to score weight matrices for time series and steady state data, respectively.

#### 2.2.b Prior knowledge and network sparsity

To enforce sparsity of *W* we use a spike-and-slab prior [23] on the edge weights: We introduce a binary latent variable, *γ*_*ij*_, for each *w*_*ij*_ indicating the presence (*γ*_*ij*_ = 1) or absence (*γ*_*ij*_ = 0) of edge *i→j*. Given *γ*_*ij*_ the spike-and-slab prior on is defined as:

$$\omega_{ij}∼\gamma_{ij}N(0,\sigma_{1}^{2})+(1-\gamma_{ij})N(0,\sigma_{2}^{2})$$ (8)

The variance σ_1_ of the slab distribution can be set sufficiently large (here: 10) in order to achieve a low bias of weight estimates for present edges. On the other hand, σ_2_ is set close to zero (σ_2_*→*0) to approximate a delta function centered at zero (the spike). The mixture coefficient γ_ij_ is drawn from a Bernouli distribution:

$$\gamma_{ij}∼Bernoulli(\rho_{ij})$$

Hence, *γ*_*ij*_ selects either the spike (if it is zero) or the slab distribution (if it is one) for *w*_*ij*_. Parameter *ρ*_*ij*_ reflects the prior probability for that. This allows us to incorporate prior knowledge in a similar way as e.g. described in [28].

#### 2.2.c Bayesian model inference via expectation propagation

Expectation propagation (EP) has been introduced by [22] as a computationally efficient approximation of full Bayesian inference. It extends the technique of moment matching [29].

Let Ө denote the set of all inferable parameters (*W*,***α***,***β***) of our model. Similar to Variational Bayesian methods, EP minimizes Kullback-Leibler (KL) divergence between the true joint distribution *p(Ө*,*D)* and some approximation, *q(Ө*,*D)*. For that purpose it is essential to factorize the joint distribution *p(Ө*,*D)*, for example as:

$$\begin{array}{l} p(\theta ,D)=p(\theta )\prod_{c\in C}^{}\frac{1}{Z_{c}}\exp\{-\sum_{t=1}^{T-1}\sum_{i=1}^{n}\sum_{r=1}^{q_{i}}{\left(y_{ir}^{c}(t+1)-\Delta_{t}\beta_{i}R_{ir}^{c}(t)-y_{ir}^{c}(t)[1-\alpha_{i}\Delta_{t}]\right)}^{2}\}\\ =\prod_{c=0}^{m}f_{c}(\theta ) \end{array}$$

with *f*_*0*_*(Ө)*: *= p(Ө)*. Each factor *f*_*c*_*(Ө)* is approximated by a multivariate Gaussian ${\tilde{f}}_{c}(\theta )$. EP then iteratively minimizes the KL-divergence $KL[p\|q]=\int p(x)\log\frac{p(x)}{q(x)}dx$ between the original distribution $p(\theta ,D)=\prod f_{c}(\theta )$ and the Gaussian approximation $q(\theta ,D)=\prod {\tilde{f}}_{c}(\theta )$. This is done using matching first moments, i.e. expectations. Notably, the EP algorithm always converges when the approximating factors are in the exponential family [22].

#### 2.2.d Implementation

**Fig 2:**
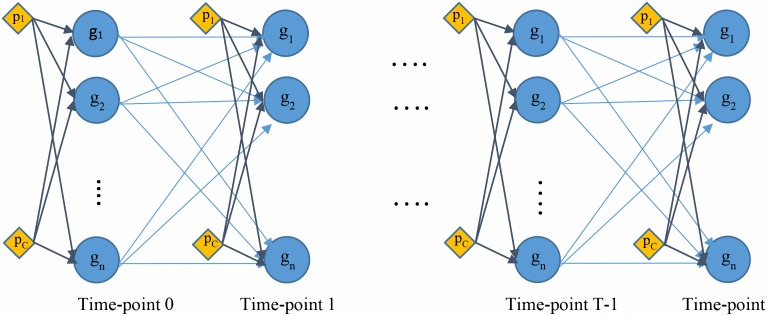
The proposed model can be interpreted as a Dynamic Bayesian Network. The network has two types of node: 1) regular nodes, demonstrated by circles, representing the genes(proteins) in the underlying biological process; 2) perturbation sources (*p*_1_, …, *p*_*C*_), represented as diamonds.

For the implementation of the EP algorithm we use microsoft Infer.NET [30], a framework for Bayesian inference on graphical models. Our proposed model can be interpreted as a special type of Dynamic Bayesian Network (DBN), connecting each network node to its parents i.e. the node values in previous time-step (Fig 2). The code of our model–written in C#–is provided in the Supplemental material (S1 Code).

The same weight matrix (or the same set of weight parameters) is used for all layers of our DBN; for example if we assume w_12_ as the weight of the edge from g_1_ at time-point 0 to g_2_ at time-point 1, the edge connecting the same two nodes from time-point 1 to time-point 2 would have same weight parameter. This implies that we assume the network structure not to change over time.

### 2.3 Dealing with non-linearity within the EP framework

The non-linear sigmoid function shown in Eq (4) yields severe convergence issues within the EP inference framework. We thus use a piece-wise approximation of the sigmoid function $g(Z)=\frac{1}{1+\exp(-Z)}$ appearing on the right hand side of Eqs (4) and (5):

$$g(\Phi (Z))=\left\{ \begin{array}{l} \Phi (Z):\;\text{if}\;\text{min}(\text{y})<\Phi (Z)<\max(y)\\ \max(y):\;\text{if}\;\Phi (Z)>\max(y)\\ \min(y):\;\text{if}\;\Phi (Z)<\min(y) \end{array}\right.$$ (9)

where *max(y)* and *min(y)* denote the maximally and minimally measured concentrations for one particular molecule (per replicate) in a certain condition over a complete time series.

Note Eq (9) provides an upper an lower bound for the concentration dependent change of each molecular in dependency of its regulators. The function *Φ* in the simplest case could be the identity function, as proposed in Bonneau, Reiss et al. (2006). In that case between the upper and lower bound the function *g* is fully linear, and deviates significantly from the original sigmoid curve if the argument *Z* is far away from 0.5. Furthermore, a linear concentration change is principally non-physiological. In order to account for these facts we thus suggest to define *Φ* in Eq (9) as a non-parametric B-spline basis expansion [31]. The B-spline expansion can be computed in a pre-processing step of our method, which maps the original time-series data into a cubic B-spline space. That means we replace each $z_{pr}^{c}$ in Eqs (4) and (5) by

$$\Phi (Z_{pr}^{c})=\sum_{k}\xi_{k}^{cpr}B_{k}^{cpr}(z_{pr}^{c})$$ (10)

and use Eq (9) as an approximation of the sigmoid function. In Eq (10) $B_{k}^{cpr}$ denotes a (cubic) B-spline basis function and the sum runs over the different spline knots *k*. After choosing an appropriate number of spline knots, functions of the form of Eq (10) can be fitted to each measured time series (on log scale). These functions can in principle be evaluated up to every desired resolution. Correspondingly, interpolated input data can now be fed to our model rather than the original raw data.

In practice we tried the following different spline interpolation methods here:

1. Smoothing B-splines, as implemented in the “smooth.spline” function in R [32]
2. Interpolated cubic B-splines, as implemented in the “spline” function in R [33]

## Results

### 3.1 Simulation studies

In order to better understand the principle behavior of our method named FBISC (Fast Bayesian Inference of Sparse Causal networks) under different conditions, we performed several simulation experiments. A rigorous comparison against competing methods is shown in later Sections.

Network topologies with 10, 50 and 100 nodes and corresponding time series data with 5, 10, 15 and 20 measurement time points were simulated via the GeneNetWeaver tool [34]. GeneNetWeaver samples random sub-networks from known large-scale *yeast* and *E*. *coli* transcriptional networks. The network topologies used in this paper are shown in the (S1 Fig). Data simulation for each of these topologies was repeated 5 times, and no perturbations were applied at first place.

**Fig 3:**
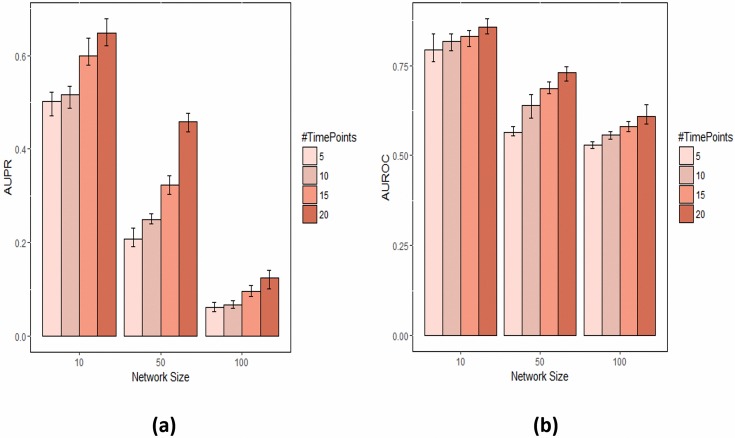
Effect of number of time points and number of network nodes on network reconstruction performance with FBISC. (a) AUPR. (b) AUROC.

The area under ROC (AUROC) and area under precision-recall curve (AUPR) are used to evaluate prediction of method. Notably, these measures are independent of a specific confidence or *p*-value cutoff. Fig 3 depicts the influence of the number of time points and the number of network nodes on reconstruction performance when using the most basic version of our method without spline interpolation (i.e. a piecewise linear approximation of the sigmoid curve). As expected, AUPR and AUROC values increase with more time points. For networks with 10 nodes and 15 time points, AUROC and AUPR reach 60% and 85%, respectively, while for networks with 100 nodes AUPR drops to 15%, but the AUROC is still close to 60%.

**Fig 4:**
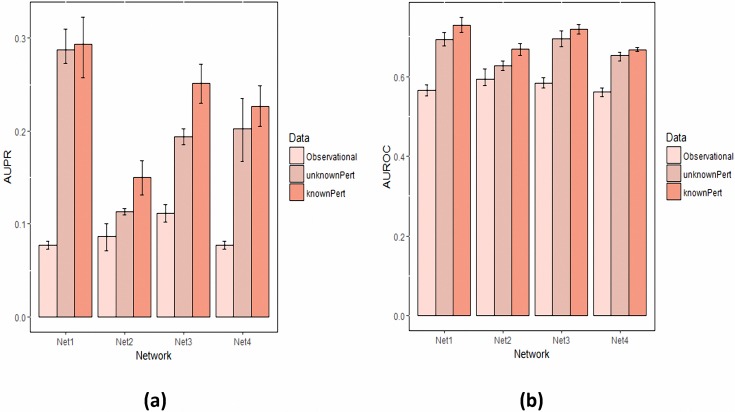
Effect of perturbation data on network reconstruction performance (a and b represent AUPR and AUROC respectively). unknownPert = targets of perturbations are not known; knownPert = targets of perturbations are fully known; observationsl = purely observational data.

Next we investigated the effect of using perturbation data with the same basic version of FBISC. For that purpose, we randomly picked 20% of the nodes of the network with 100 nodes and each of them affected by a different perturbation. Perturbations were assumed to represent a constant signal over time, and ten time points were simulated for time courses of each network node. We compared three situations 1) targets of perturbations are fully known; 2) targets of perturbations are unknown; 3) purely observational data. Fig 4 represents our results for four network structures for each of these situations.

As indicated by Fig 4 perturbation data generally increased AUPR and AUROC compared to purely observational data dramatically: AUPR was at least twice as high than with purely observational data, and the AUROC increased by more than 10%. If targets of individual perturbations were fully known to the algorithm (i.e. the prior probability for edges connecting perturbations to their targets was one) AUPR and AUROC values were on average ~5% higher than in the case were targets of perturbations were unknown.

**Fig 5:**
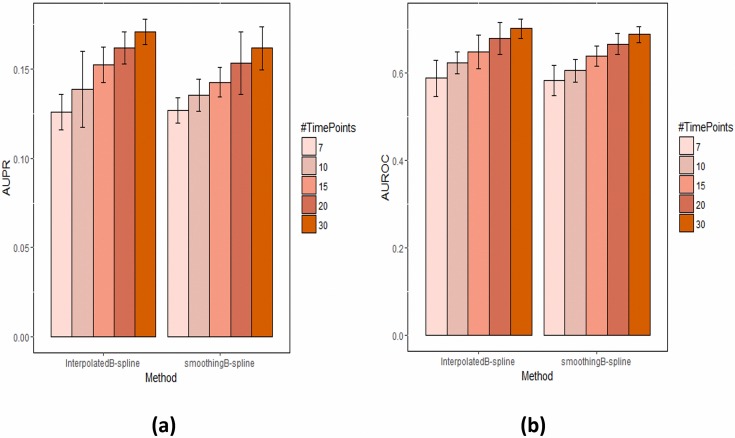
Effect of using spline interpolation and smoothing spline. The plot shows the AUPR and AUROC (a and b respectively) as a function of an increasing number of interpolated measurement time points. 7 time points corresponds to the original data in combination with a piecewise linear approximation of the sigmoid curve.

In the last experiment we compared the different spline methods discussed in Section 2.3 against each other and against the piecewise linear approximation of the sigmoid curve. This was done for the network with 100 nodes and 7 simulated measurement time points. No perturbations were simulated at this point. The results shown in Fig 5 indicate a significant increase of AUPR and AUROC with both spline techniques and increasing the number of interpolated measurement time points. At the same time the difference between B-spline interpolation and smooting B-splines was marginal.

In conclusion, our simulations demonstrate that our method can successfully exploit perturbation information and profits from spline interpolated time series data. Furthermore, reconstruction performance is expected to be relatively robust, even if large networks are estimated.

### 3.2 Evaluation on DREAM challenge data and comparison against competing methods

We downloaded data from the DREAM4 [35] and DREAM8 [36, 37] challenges for the further evaluation of our approach and comparison to competing methods (see http://dreamchallenges.org/project-list/closed/). The gold standard networks provided in these data are used for evaluation. DREAM4 provides simulated data for five networks of size 10 nodes and five networks of size 100 nodes. For each network perturbation time series and steady state data were retrieved. Time series data comprise 21 time points (t_0_ = 0 to t_20_ = 1000) reflecting measurements of each network node. Perturbations are always applied at time 0 and removed at time 500. Information regarding to the exact targets of perturbations are not available. Each time series is measured with 5 replicates for the 10-node network and 10 replicates for the 100-node network. Each replicate represents a perturbation experiment in which different nodes (about one-third of the network) are perturbed.

In addition to time series different kinds of steady state data are available from DREAM4. Here we employed knock-out, knock-down, and multifactorial perturbation data. Knock-out and knock-down data reflect steady state measurements of all network nodes after perturbation of exactly one known node. Multifactorial data corresponds to combinatorial perturbations of unknown nodes in each experiment. No replicate measurements are available for steady state data.

DREAM8 provides experimental data of a signaling network with 20 nodes at 11 time points. Here the perturbations correspond to compound treatments. Exact concentrations of perturbation sources are not given but specified with qualitative values (high/low). Following Young et al. [38] we normalized each measured time series by subtracting its mean.

We compared FBISC with ScanBMA [38], iBMA [39], LASSO [40], ebdnet (Empirical Bayes Estimation of Dynamic Bayesian Networks [41], ARACNE [42], and CLR [43]. As opposed to the first three methods LASSO, ARACNE and CLR are not per se designed for time series data. In order to adapt these methods to our situation we considered for each network gene of interest all other genes as potential regulators. For each potential regulator its measurements excluding the last time point were used. We then asked, which subset of regulators could predict the measured time series of the network gene of interest excluding the first time point. This estimation procedure was repeated for all network genes.

Notably, information about perturbations was included into all methods competing with our FBISC approach. This was done by adding perturbations as additional potential “regulators” of each node (similar to the way that FBISC treats perturbations). In case that perturbation targets were known, perturbations were only considered as potential regulators of directly targeted nodes.

All tested network inference algorithms produce either a confidence measure (FBISC, ScanBMA, iBMA) or a *p*-value (ebdnet, CLR, ARACNE) for each possible edge. Correspondingly, AUROC and AUPR are used to evaluate prediction performances of methods. Notably, these measures are independent of a specific confidence or p-value cutoff.

### 3.3 Results for DREAM4 data

Using the above described time series perturbation data we compared results obtained by our method with the ones reported in Young et al. [38]. Results generated by our method are shown in the last two rows of Table 1, indicating a higher AUROC/AUPR for 10-gene and higher AUPR for 100-gene networks than competing methods, specifically when using the B-spline method. ROC and PR curves regarding DREAM4 networks of size 100 are provided in S2 Fig.

**Table 1:** Average performance results based on DREAM4 10-gene and 100-gene networks (time series data). FBISC results are shown in the last two rows; other results were taken from Young et al. (2014). FBISC-linear corresponds to the piecewise linear approximation of the sigmoid curve. FBISC-B-spline is applied with 20 and 100 interpolated time points for the 10 and 100-gene networks, respectively.

Method	AUROC (10-gene network)	AUPRC (10-gene network)	AUROC (100-gene network)	AUPRC (100-gene network)
**LASSO**	0.731	0.487	0.643	0.073
**Ebdnet**	0.704	0.438	0.643	0.043
**ARACNE**	0.668	0.388	0.589	0.106
**CLR**	0.681	0.397	**0.699**	0.123
**ScanBMA**	0.74	0.505	0.657	0.101
**FBISC-linear**	0.757	0.486	0.643	**0.161**
**FBISC-B-spline**	**0.810**	**0.510**	0.650	0.122

Next we compared our method to the competing approaches on the basis of various kinds of steady state data (Table 2), showing a clear improvement compared to ARACNE, LASSO and CLR (which are the only competing methods using steady state data) in all cases. As expected, AUROC and AUPR values for steady state data are typically a bit below those observed for time series data. However, FBISC using knock-out data could still achieve an AUROC of 70% for the 100-gene network.

**Table 2:** Average performance results based on DREAM4 10-gene and 100-gene networks with steady state knock-down, knock-out, and multifactorial data. Notably, FBISC is only applicable without spline interpolation in these situations. For the 10-gene networks LASSO failed due to low number of available samples and is thus not shown.

Method	Type of perturbation	AUROC (10-gene network)	AUPR (10-gene network	AUROC (100-gene network)	AUPR (100-gene network
**ARACNE**	Knock-down	0.503	0.200	0.525	0.076
**LASSO**	Knock-down	-	-	0.521	0.074
**CLR**	Knock-down	0.562	0.219	0.564	0.085
**FBISC**	**Knock-down**	**0.629**	**0.246**	**0.607**	**0.091**
**ARACNE**	Knock-out	0.563	0.247	0.541	0.087
**LASSO**	Knock-out	-	-	0.549	0.107
**CLR**	Knock-out	0.603	0.261	0.594	0.097
**FBISC**	**Knock-out**	**0.678**	**0.281**	**0.709**	**0.175**
**ARACNE**	Multifactorial	0.644	0.317	0.493	0.057
**LASSO**	Multifactorial	-	-	0.499	0.058
**CLR**	Multifactorial	0.652	0.310	0.495	0.056
**FBISC**	**Multifactorial**	**0.657**	**0.268**	**0.524**	**0.060**

### 3.4 Results for DREAM8 data

Next, we focused on the DREAM8 challenge data. In contrast to before, results for this dataset were obtained by our own implementation of competing methods. More specifically, we used R package NetworkBMA [44] for ScanBMA, minet [45] for ARACNE and CLR, ebdbnet [41], and glmnet [46] for LASSO. Results are presented in Table 3, indicating a slightly better AUROC than the best competing approach (CLR).

**Table 3:** Results on DREAM8 signaling data. FBISC-linear corresponds to the piecewise linear approximation of the sigmoid curve.

Method	AUROC	AUPR
**iBMA**	0.488	0.238
**ARACNE**	0.562	0.321
**LASSO**	0.545	0.326
**ebdnet**	0.493	0.257
**CLR**	0.626	**0.381**
**ScanBMA**	0.47	0.242
**FBISC-linear**	0.618	0.346
**FBISC-B-spline (30 time points)**	**0.643**	0.342

### 3.5 Effect of incorporating prior knowledge

**Fig 6:**
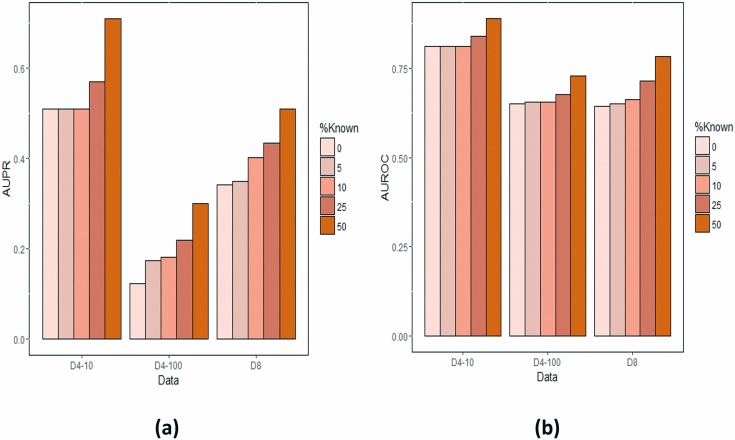
Effect of including prior knowledge into FBISC for DREAM; AUPR and AUROC represented in a and b respectively. (10 and 100-node networks: D4-10 and D4-100) and DREAM8 (D8) data. Shown are the AUPR and AUROC for FBISC after adding a varying percentage of true edges with 90% confidence.

Next we tested, in how far the previously presented results would change in dependency of prior knowledge. Only time series data were used at this point. Following the approach used by Praveen et al. [47] we considered 0, 5, 10, 25 and 50 percent of true network edges to be known with probability of 90%, and the FBISC-B-spline method with the same setting reported in Sections 3.3 and 3.4 was used. The results of this experiment (Fig 6) show an increase of AUROC and AUPR as fractions of confidently known edges increase. Notably, with 50% known edges we could achieve an AUROC of close to 75% for the 100 gene network from the DREAM4 challenge and about the same performance for the DREAM8 challenge network.

### 3.6 Run time and parallelization

FBISC is a Bayesian approach. Frequentist methods like CLR and ARACNE are based on mutual information and conceptually far simpler. They are thus computationally comparably cheap. From the practical point of view, a question is thus, how the computing time of FBISC compares to competing methods.

The run time of ScanBMA depends on the number of potential parents (*nvar*) per node. Here we tested ScanBMA with *nvar* = 10 and *nvar* = 20 (for larger values of *nvar* the run time increases dramatically). Results for our method compared to the competing approaches considered in this paper are reported in Table 4, indicating a good computational scalability of our FBISC approach. Notably, our algorithm can be parallelized, because the inference problem can be solved independently for each network node. This allows for an additional gain in computation time. Corresponding results shown in Table 4 (named FBISC parallel) refer to the use of 2 cores (Intel® Core™ i5-5257U dual core processor with 4 parallel threads). More cores would reduce calculation time even more. For DREAM8 and DREAM4 networks of size 100 there is speed up by factor 2 due to parallelization (Table 4). At the same time, the memory use was rather modest and allowed to run all computations on a standard laptop computer. Overall, the Bayesian inference scheme used in FBISC thus seems to be applicable even for large networks.

**Table 4:** Total run time (in seconds) on DREAM4 (10 and 100-node networks) and DREAM8 data.

Method	DREAM4 (10-gene network)	DREAM4 (100-gene network)	DREAM8
**FBISC100 EP iter**	7.349	186.639	71.699
**FBISC 20 EP iter**	3.901	59.930	20.047
**FBISC parallel (20 iter)**	3.469	30.704	10.250
**ScanBMA (nvar = 10)**	3.978	60.547	16.266
**ScanBMA (nvar = 20)**	4.746	4305.107	8315.338
**LASSO**	1.05	38.4	2.34
**Ebdbnet**	0.044	25.529	0.31
**CLR**	0.002	0.01	0.004
**ARACNE**	0.002	0.013	0.124

## Discussion

We proposed a Bayesian approach for computationally efficient inference of large scale molecular networks from complex perturbation data. Our FBISC method is highly flexible and applicable even in situations where the exact targets of perturbations are unknown (such as stress experiments), which is frequently the case in biology. A further strength is that we consider perturbations themselves as time dependent, as e.g. reflected in the DREAM4 data. FBISC uses a biochemically inspired model to describe the non-linear dynamical behavior of molecular networks and integrates this description into a graphical modeling framework. This allows for the application of efficient approximate inference schemes, such as expectation propagation. Notably, the output of our method is a posterior distribution over edge weights, which accounts for the unavoidable uncertainty of any network inference.

We enforced sparsity of inferred networks in form of a spike and slab prior. This type of prior forces many edge weights to exactly zero and naturally allows for the integration of prior background knowledge, which demonstrated useful in our results. Altogether we see the combination of a highly flexible modeling framework (reflected by non-linear dynamics, arbitrary complex perturbation schemes and probabilistic integration of prior knowledge), which is applicable to time series as well as steady state data and uses computationally scalable Bayesian inference as differentiation of FBISC to existing techniques. The advantage for the user lies in a unified method, which allows for automatically adapting literature derived network information to experimental data and produces confidence measures. Our results showed an attractive prediction performance of our method. We thus believe that our proposed FBISC method is an attractive alternative to existing methods to learn causal network structures from complex perturbation data. The C# code of our method is included in the supplements of this paper (S1 Code).

## Supporting information

S1 Code Code for FBISC inference model. (c# infer.net framework).
(CS) Click here for additional data file.

S1 Fig Network structures used in simulation section. (generated by geneNetWeaver).
(DOCX)

S2 Fig ROC and PR curves for the rest of DREAM4 size 100 networks number. (DOCX)

## References

[1] Albert R. Network inference, analysis, and modeling in systems biology. The Plant Cell. 2007;19(11):3327–38. 10.1105/tpc.107.054700 18055607 PMC2174897

[2] Fire A, Xu S, Montgomery MK, Kostas SA, Driver SE, Mello CC. Potent and specific genetic interference by double-stranded RNA in Caenorhabditis elegans. nature. 1998;391(6669):806–11. 10.1038/35888 9486653

[3] Sander JD, Joung JK. CRISPR-Cas systems for editing, regulating and targeting genomes. Nature biotechnology. 2014;32(4):347–55. 10.1038/nbt.2842 24584096 PMC4022601

[4] Wagner A. How to reconstruct a large genetic network from n gene perturbations in fewer than n2 easy steps. Bioinformatics. 2001;17(12):1183–97. 11751227 10.1093/bioinformatics/17.12.1183

[5] Tresch A, Beissbarth T, Sültmann H, Kuner R, Poustka A, Buness A. Discrimination of direct and indirect interactions in a network of regulatory effects. Journal of Computational Biology. 2007;14(9):1217–28. 10.1089/cmb.2007.0085 17990974

[6] Klamt S, Flassig RJ, Sundmacher K. TRANSWESD: inferring cellular networks with transitive reduction. Bioinformatics. 2010;26(17):2160–8. 10.1093/bioinformatics/btq342 20605927 PMC2922889

[7] Pe’er D. Bayesian network analysis of signaling networks: a primer. Sci STKE. 2005;281:l4. 10.1126/stke.2812005pl4 15855409

[8] Pe’er D, Regev A, Elidan G, Friedman N. Inferring subnetworks from perturbed expression profiles. Bioinformatics. 2001;17(suppl 1):S215–S24. 11473012 10.1093/bioinformatics/17.suppl_1.s215

[9] Sachs K, Perez O, Pe'er D, Lauffenburger DA, Nolan GP. Causal protein-signaling networks derived from multiparameter single-cell data. Science. 2005;308(5721):523–9. 10.1126/science.1105809 15845847

[10] Maathuis MH, Colombo D, Kalisch M, Bühlmann P. Predicting causal effects in large-scale systems from observational data. Nature Methods. 2010;7(4):247–8. 10.1038/nmeth0410-247 20354511

[11] Mazloomian A, Beigy H. Inferring signaling pathways using interventional data. Intelligent Data Analysis. 2013;17(2):295–308.

[12] Yavari F, Towhidkhah F, Gharibzadeh S, Khanteymoori A, Homayounpour M, editors. Modeling large-scale gene regulatory networks using gene ontology-based clustering and dynamic bayesian networks. in 2008 2nd International Conference on Bioinformatics and Biomedical Engineering. IEEE. 2008. p. 297–300.

[13] Gat-Viks I, Tanay A, Raijman D, Shamir R. A probabilistic methodology for integrating knowledge and experiments on biological networks. Journal of Computational Biology. 2006;13(2):165–81. 10.1089/cmb.2006.13.165 16597233

[14] Van Driessche N, Demsar J, Booth EO, Hill P, Juvan P, Zupan B, et al Epistasis analysis with global transcriptional phenotypes. Nature genetics. 2005;37(5):471–7. 10.1038/ng1545 15821735

[15] Nelander S, Wang W, Nilsson B, She QB, Pratilas C, Rosen N, et al Models from experiments: combinatorial drug perturbations of cancer cells. Molecular systems biology. 2008;4(1):216. 18766176 10.1038/msb.2008.53 PMC2564730

[16] Molinelli EJ, Korkut A, Wang W, Miller ML, Gauthier NP, Jing X, et al Perturbation biology: inferring signaling networks in cellular systems. PLoS Comput Biol. 2013;9(12):e1003290 10.1371/journal.pcbi.1003290 24367245 PMC3868523

[17] Markowetz F, Kostka D, Troyanskaya OG, Spang R. Nested effects models for high-dimensional phenotyping screens. Bioinformatics. 2007;23(13):i305–i12. 10.1093/bioinformatics/btm178 17646311

[18] Fröhlich H, Tresch A, Beissbarth T. Nested effects models for learning signaling networks from perturbation data. Biometrical Journal. 2009;51(2):304–23. 10.1002/bimj.200800185 19358219

[19] Bansal M, Della Gatta G, Di Bernardo D. Inference of gene regulatory networks and compound mode of action from time course gene expression profiles. Bioinformatics. 2006;22(7):815–22. 10.1093/bioinformatics/btl003 16418235

[20] Molinelli EJ, Korkut A, Wang W, Miller ML, Gauthier NP, Jing X, et al Perturbation biology: inferring signaling networks in cellular systems. PLoS Comput Biol. 2013 9(12): p. e1003290 10.1371/journal.pcbi.1003290 24367245 PMC3868523

[21] Yip KY, Alexander RP, Yan K-K, Gerstein M. Improved reconstruction of in silico gene regulatory networks by integrating knockout and perturbation data. PloS one. 2010;5(1):e8121 10.1371/journal.pone.0008121 20126643 PMC2811182

[22] Minka TP, editor Expectation propagation for approximate Bayesian inference Proceedings of the Seventeenth conference on Uncertainty in artificial intelligence; 2001: Morgan Kaufmann Publishers Inc 2001 p. 362–369.

[23] George EI, McCulloch RE. Approaches for Bayesian variable selection. Statistica sinica. 1997;7(2):339–73.

[24] Bonneau R, Reiss DJ, Shannon P, Facciotti M, Hood L, Baliga NS, et al The Inferelator: an algorithm for learning parsimonious regulatory networks from systems-biology data sets de novo. Genome biology. 2006;7(5):R36 10.1186/gb-2006-7-5-r36 16686963 PMC1779511

[25] Eaton D, Murphy KP, editors. Exact Bayesian structure learning from uncertain interventions. AISTATS; 2007. p. 107–114.

[26] De Jong H. Modeling and simulation of genetic regulatory systems: a literature review. Journal of computational biology. 2002;9(1):67–103. 10.1089/10665270252833208 11911796

[27] Zacher B, Abnaof K, Gade S, Younesi E, Tresch A, Fröhlich H. Joint Bayesian inference of condition-specific miRNA and transcription factor activities from combined gene and microRNA expression data. Bioinformatics. 2012;28(13):1714–20. 10.1093/bioinformatics/bts257 22563068

[28] Fröhlich H. biRte: Bayesian inference of context-specific regulator activities and transcriptional networks. Bioinformatics. 2015;31(20):3290–8. 10.1093/bioinformatics/btv379 26112290

[29] Lauritzen SL. Propagation of probabilities, means, and variances in mixed graphical association models. Journal of the American Statistical Association. 1992;87(420):1098–108.

[30] Minka T, Winn J, Guiver J, Knowles D. Infer.NET 2.5. Microsoft Research Cambridge. 2012.

[31] Morrissey ER, Juárez MA, Denby KJ, Burroughs NJ. Inferring the time-invariant topology of a nonlinear sparse gene regulatory network using fully Bayesian spline autoregression. Biostatistics. 2011;12(4):682–94. 10.1093/biostatistics/kxr009 21551122

[32] Chambers J, Hastie T. Statistical Models in S (Wadsworth & Brooks, Pacific Grove, CA). 1992.

[33] Forsythe GE, Moler CB, Malcolm MA. Computer methods for mathematical computations. 1977.

[34] Schaffter T, Marbach D, Floreano D. GeneNetWeaver: in silico benchmark generation and performance profiling of network inference methods. Bioinformatics. 2011;27(16):2263–70. 10.1093/bioinformatics/btr373 21697125

[35] DREAM4 In silico network challenge. Available from: https://www.synapse.org/#!Synapse:syn3049712/wiki/74628.

[36] Hill S. HPN-DREAM Breast Cancer Challenge. Nat BioTech. 2015.

[37] Hill SM, Heiser LM, Cokelaer T, Unger M, Nesser NK, Carlin DE, et al Inferring causal molecular networks: empirical assessment through a community-based effort. Nature methods. 2016;13(4):310–8. 10.1038/nmeth.3773 26901648 PMC4854847

[38] Young WC, Raftery AE, Yeung KY. Fast Bayesian inference for gene regulatory networks using ScanBMA. BMC systems biology. 2014;8(1):47. 24742092 10.1186/1752-0509-8-47 PMC4006459

[39] Lo K, Raftery AE, Dombek KM, Zhu J, Schadt EE, Bumgarner RE, et al Integrating external biological knowledge in the construction of regulatory networks from time-series expression data. BMC systems biology. 2012;6(1):1. 22898396 10.1186/1752-0509-6-101 PMC3465231

[40] Tibshirani R. Regression shrinkage and selection via the lasso. Journal of the Royal Statistical Society Series B (Methodological). 1996:267–88.

[41] Rau A, Jaffrézic F, Foulley J-L, Doerge RW. An empirical Bayesian method for estimating biological networks from temporal microarray data. Statistical Applications in Genetics and Molecular Biology. 2010;9(1). 10.2202/1544-6115.1513 20196759

[42] Margolin AA, Nemenman I, Basso K, Wiggins C, Stolovitzky G, Favera RD, et al ARACNE: an algorithm for the reconstruction of gene regulatory networks in a mammalian cellular context. BMC bioinformatics. 2006;7(Suppl 1):S7. 10.1186/1471-2105-7-S1-S7 PMC1810318 16723010

[43] Faith JJ, Hayete B, Thaden JT, Mogno I, Wierzbowski J, Cottarel G, et al Large-scale mapping and validation of Escherichia coli transcriptional regulation from a compendium of expression profiles. PLoS biol. 2007;5(1):e8 10.1371/journal.pbio.0050008 17214507 PMC1764438

[44] Fraley C, Yeung K, Raftery A. networkBMA: Regression-based network inference using BMA, 2012. R package distributed through Bioconductor.

[45] Meyer PE, Lafitte F, Bontempi G. minet: AR/Bioconductor package for inferring large transcriptional networks using mutual information. BMC bioinformatics. 2008;9(1):1. 18959772 10.1186/1471-2105-9-461 PMC2630331

[46] Friedman J, Hastie T, Tibshirani R. Regularization paths for generalized linear models via coordinate descent. Journal of statistical software. 2010;33(1):1 20808728 PMC2929880

[47] Praveen P, Fröhlich H. Boosting probabilistic graphical model inference by incorporating prior knowledge from multiple sources. PloS one. 2013;8(6):e67410 10.1371/journal.pone.0067410 23826291 PMC3691143

