# Supplementary material for: Expectation propagation for large scale Bayesian inference of non-linear molecular networks from perturbation data
Source: PLoS One. 2017 Feb 6;12(2):e0171240. doi: 10.1371/journal.pone.0171240 (PMC5293552; doi:10.1371/journal.pone.0171240)
Supplement: S1 Fig — (generated by geneNetWeaver). (DOCX) [file pone.0171240.s002.docx]

**S1 Fig. Network structures used in simulation section (generated by geneNetWeaver [1] ).** a) size 10 b) size 50 c,d) size 100


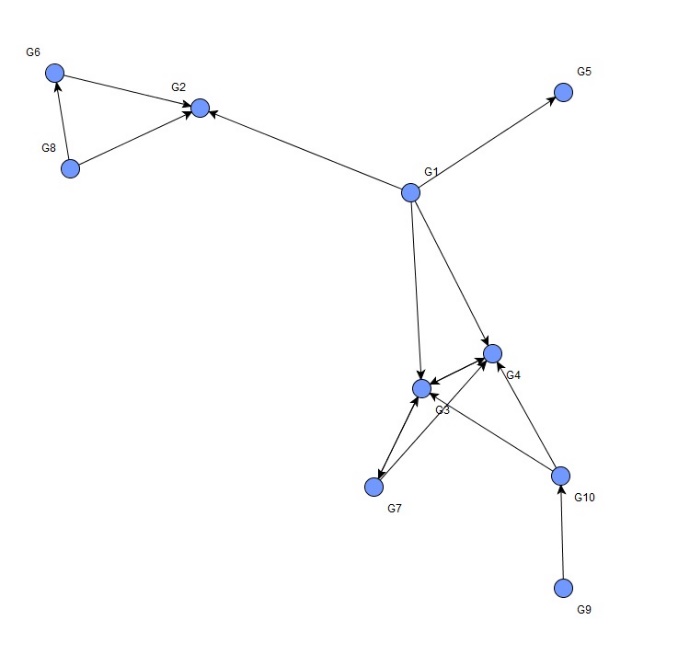


(a)


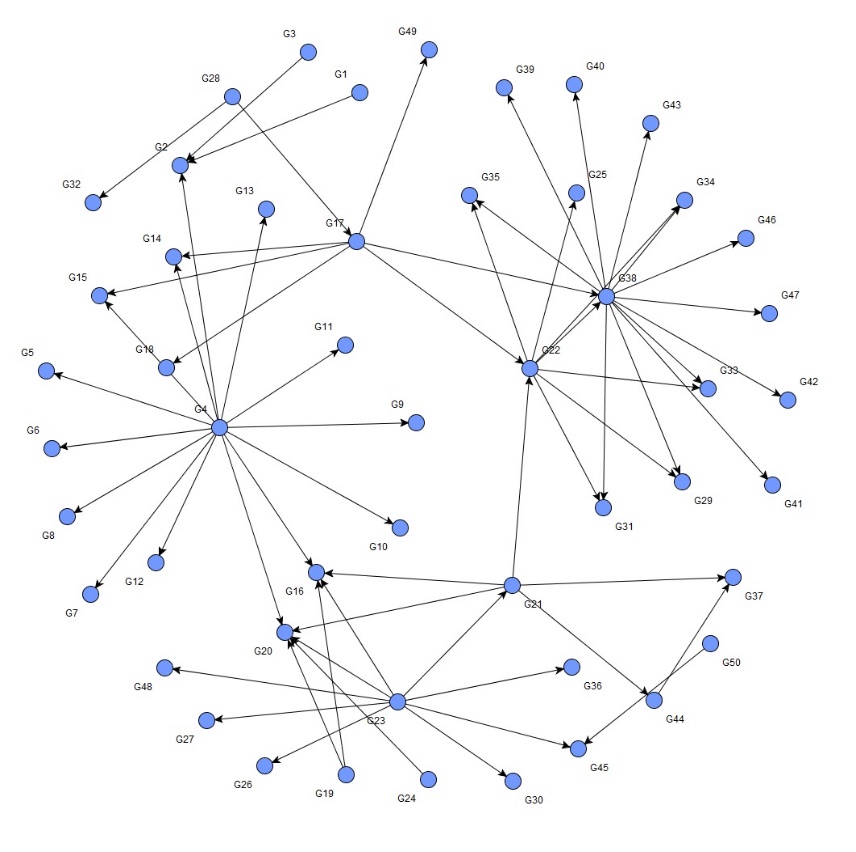


(b)


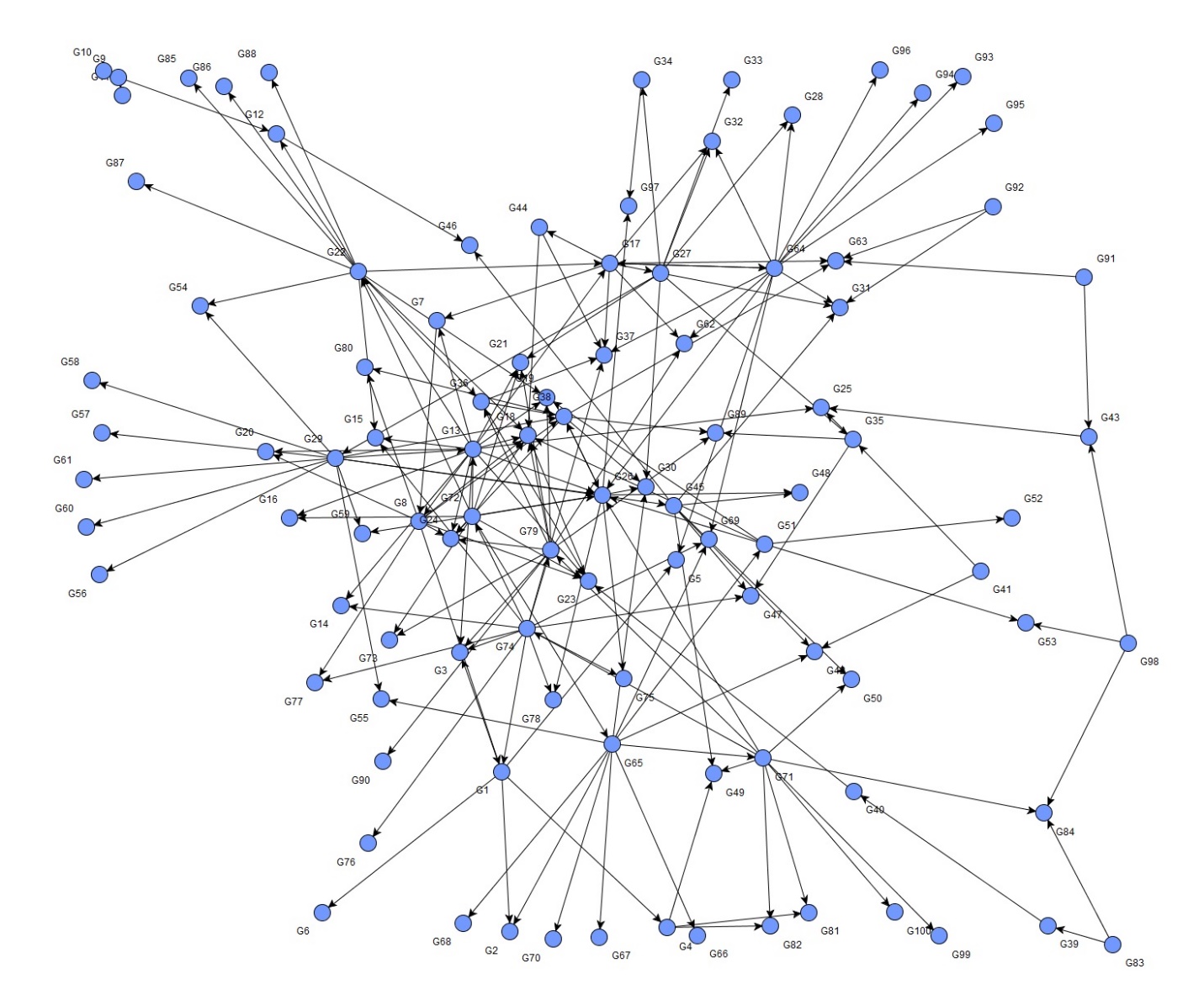


(c)


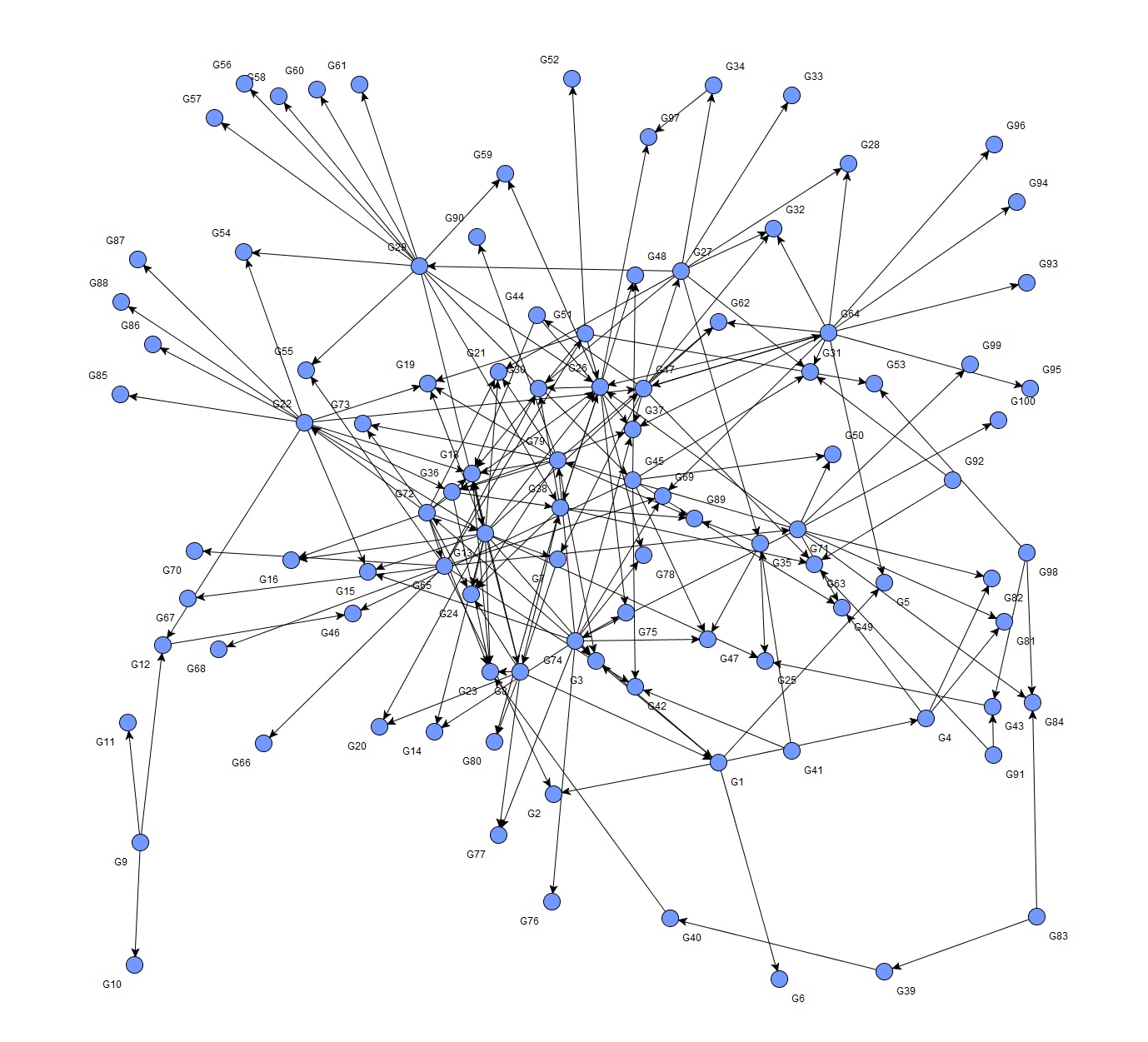


(d)

**References**

1. Schaffter, T., D. Marbach, and D. Floreano, *GeneNetWeaver: in silico benchmark generation and performance profiling of network inference methods.* Bioinformatics, 2011. **27**(16): p. 2263-2270.
