# Supplementary figures and images for: Expectation propagation for large scale Bayesian inference of non-linear molecular networks from perturbation data
Source: PLoS One. 2017 Feb 6;12(2):e0171240. doi: 10.1371/journal.pone.0171240 (PMC5293552; doi:10.1371/journal.pone.0171240)

**S2 Fig. ROC and PR curves for the rest of DREAM4 size 100 networks number (a-d)**

a)


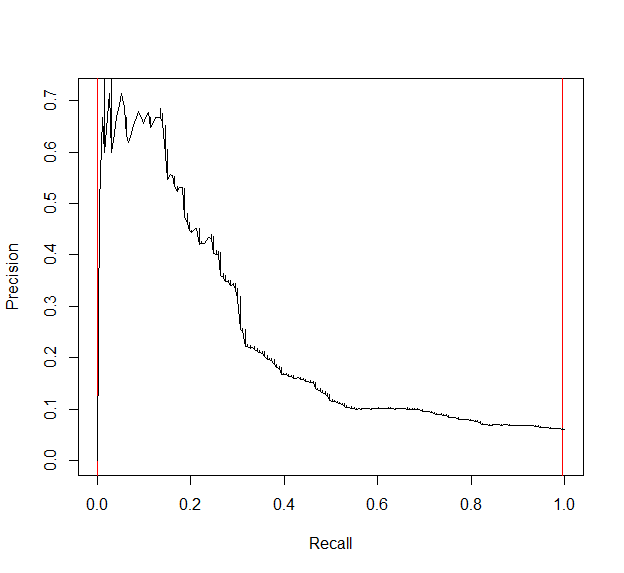

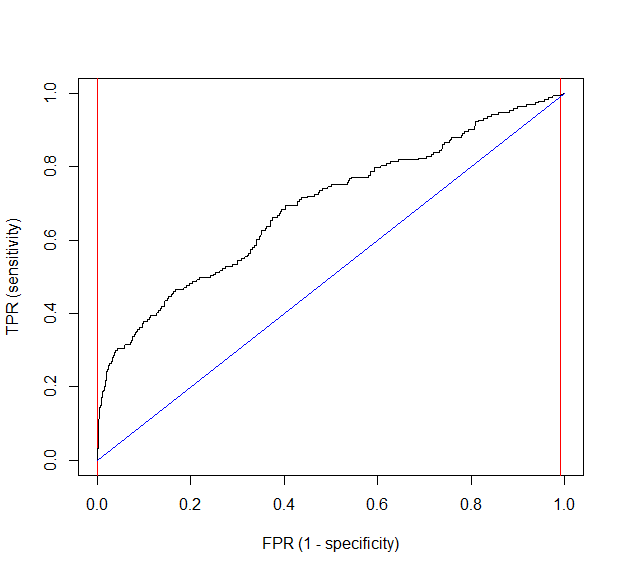


b)


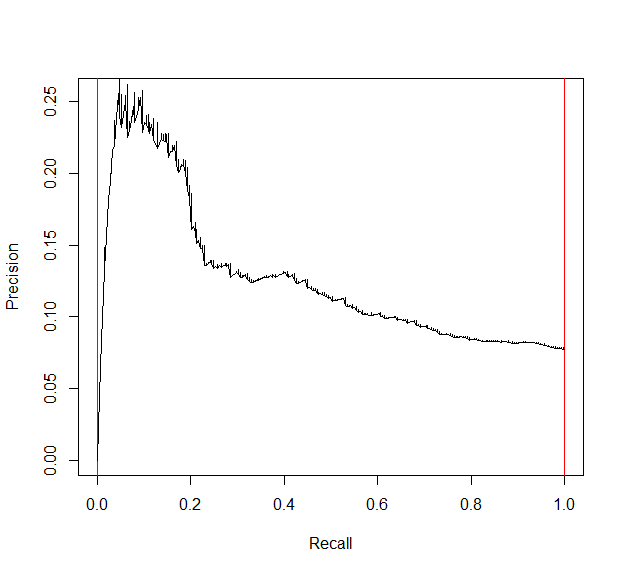

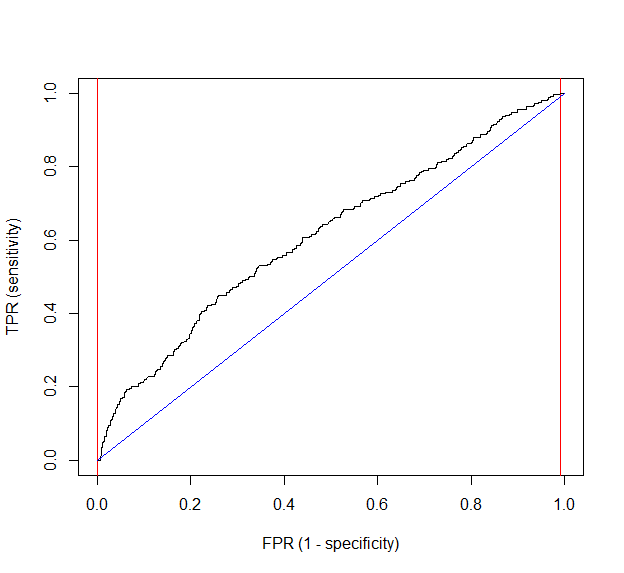


c)


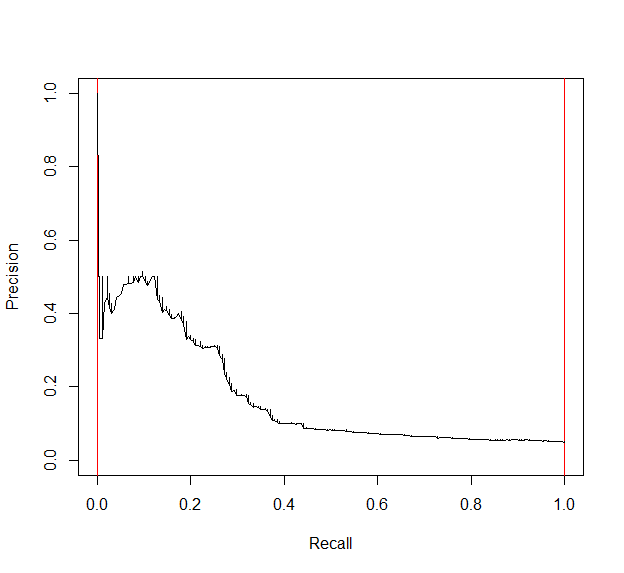

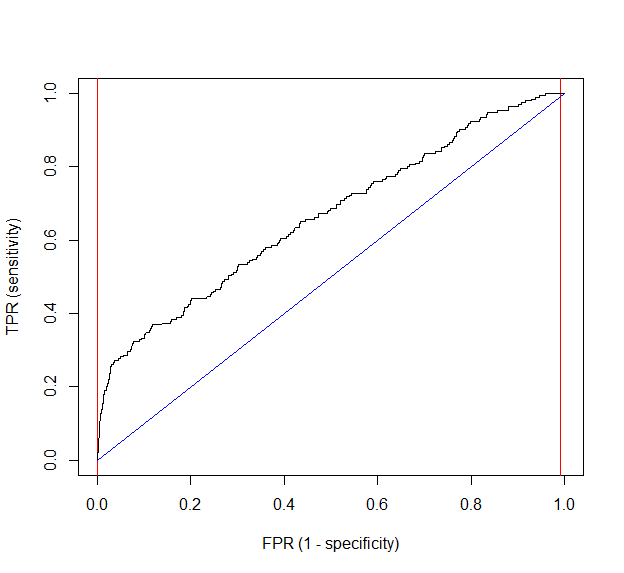


d)


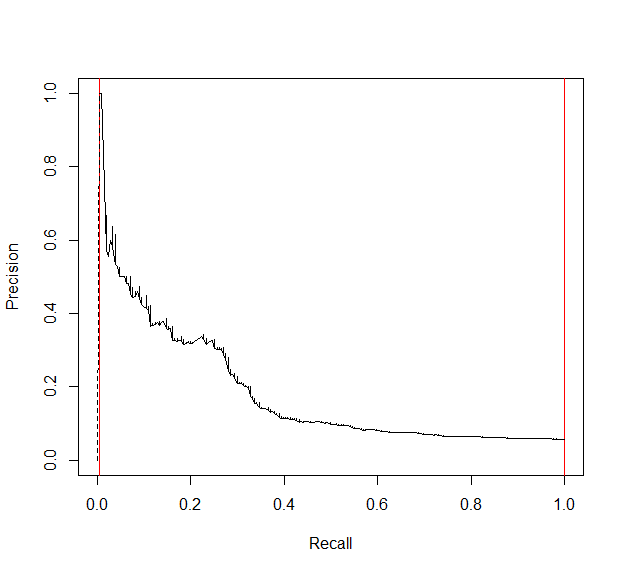

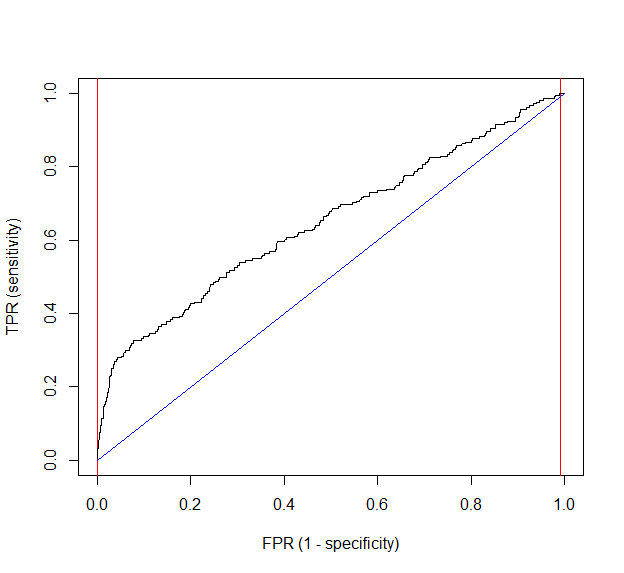


e)


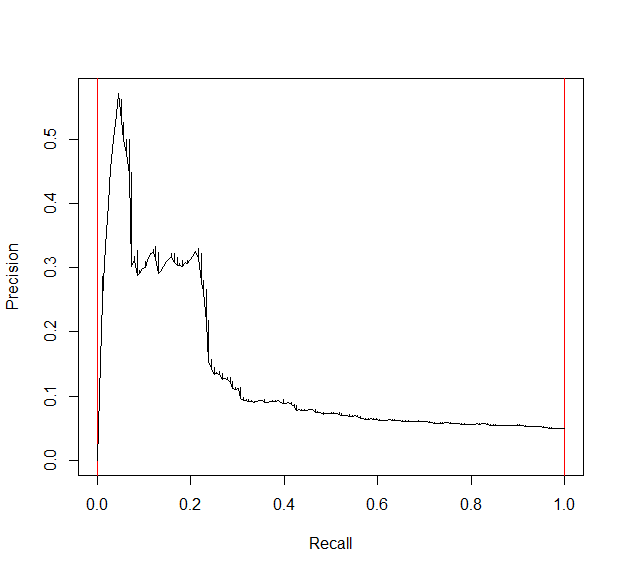

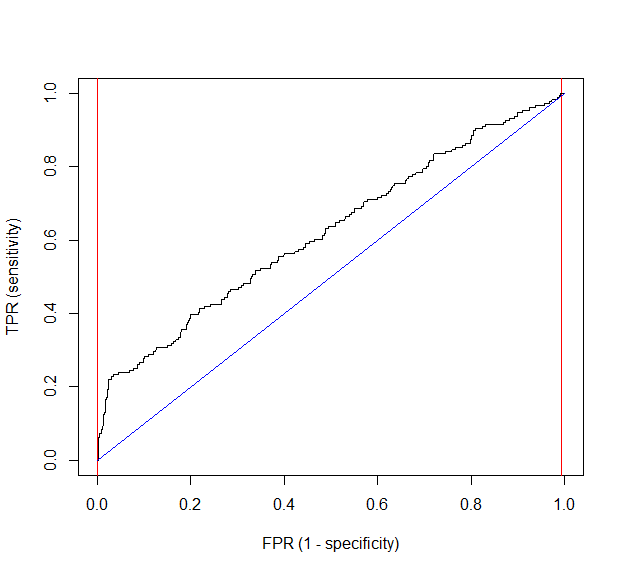

Supplement: S2 Fig — (DOCX) [file pone.0171240.s003.docx]
